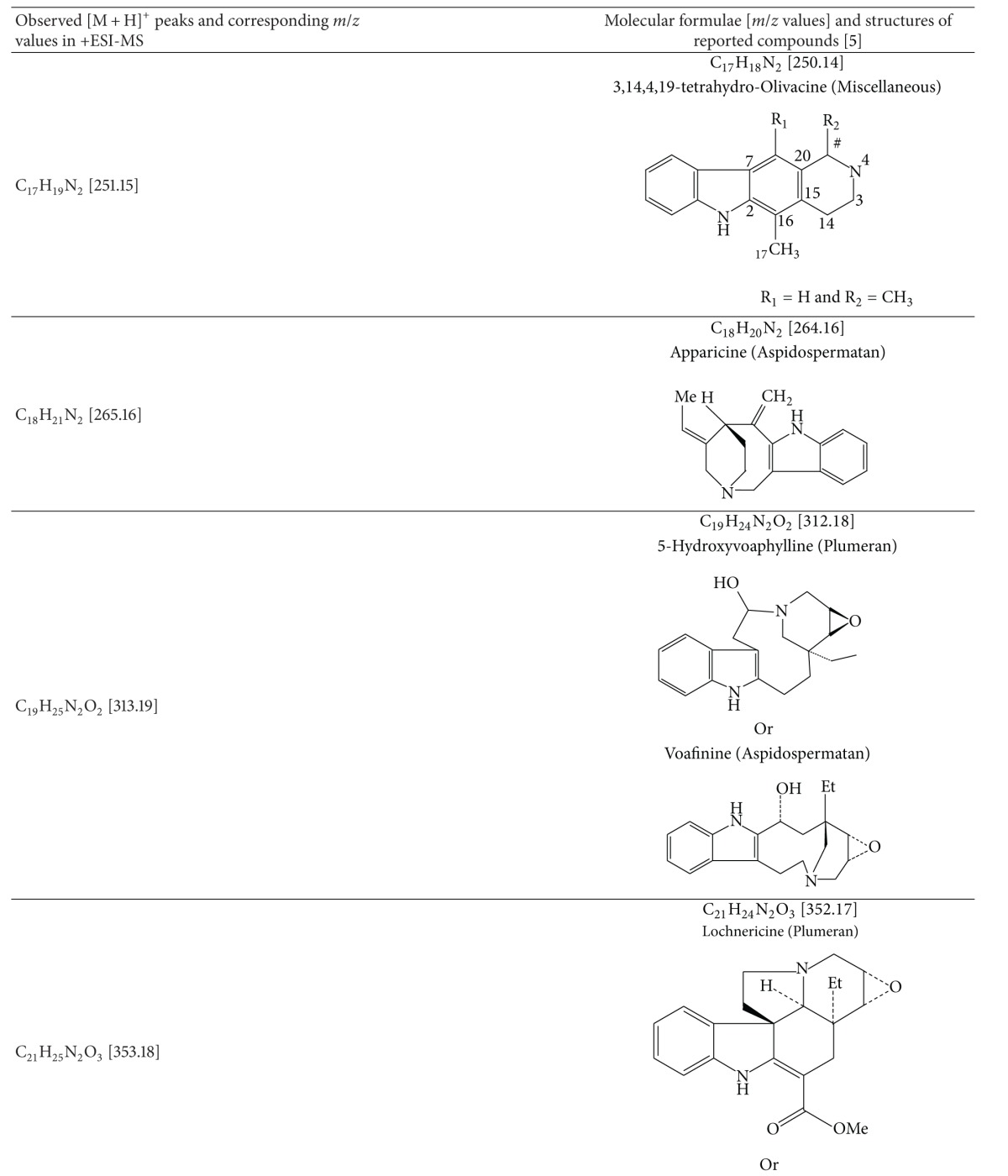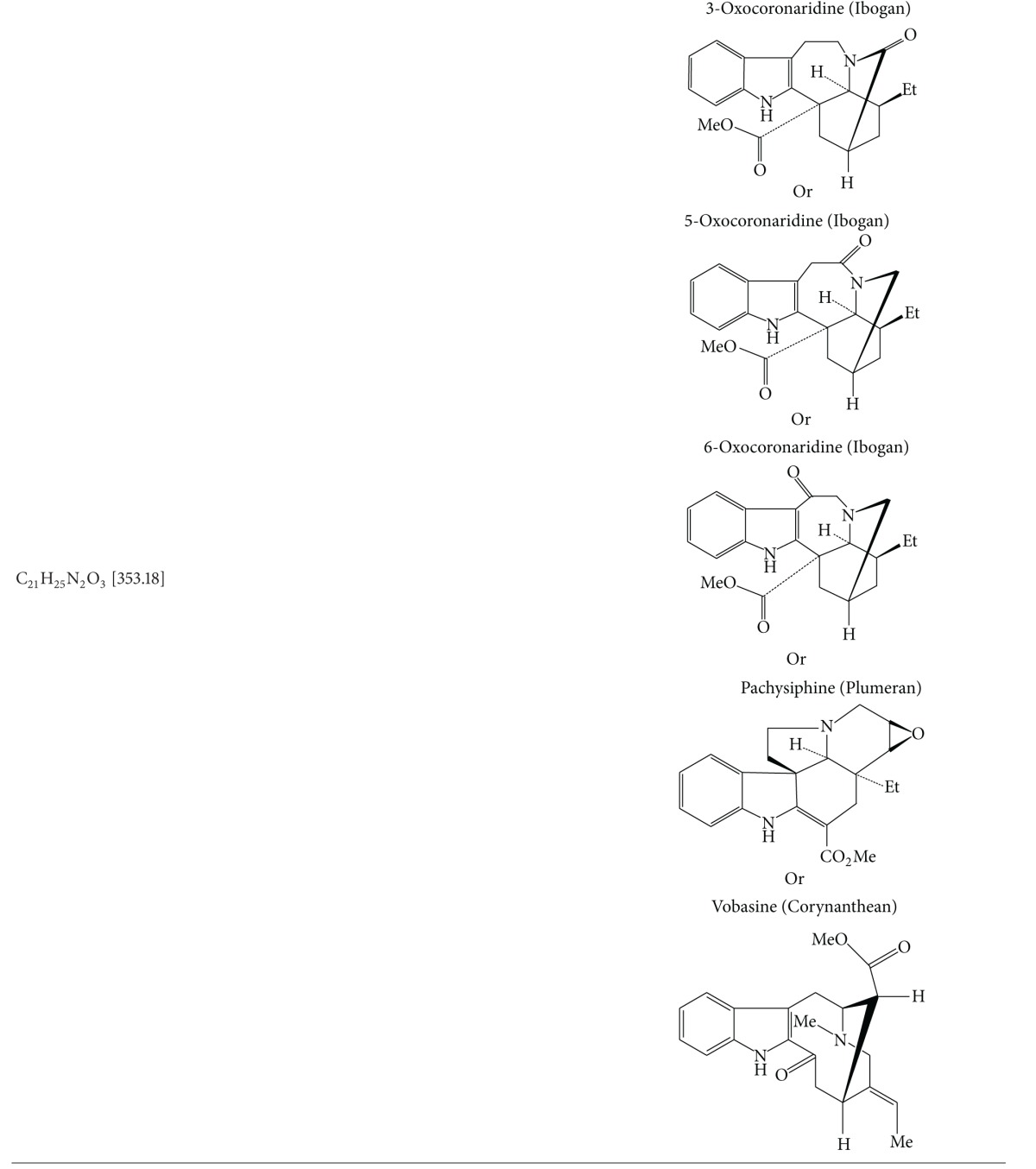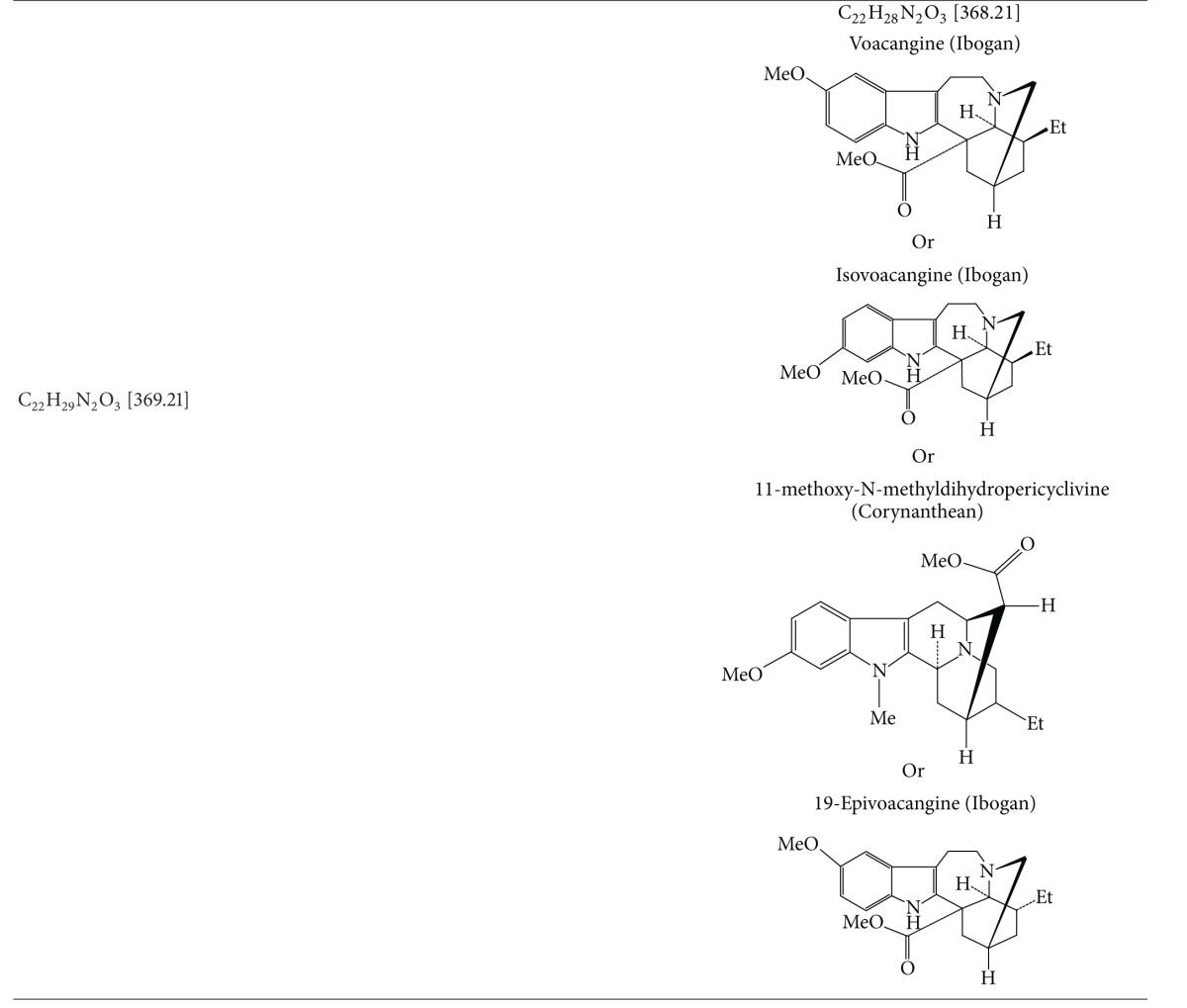# Prostaglandin Analogous and Antioxidant Activity Mediated Gastroprotective Action of *Tabernaemontana divaricata * (L.) R. Br. Flower Methanolic Extract against Chemically Induced Gastric Ulcers in Rats

**DOI:** 10.1155/2013/185476

**Published:** 2013-10-29

**Authors:** Mohammed Safwan Ali Khan, Abdul Manan Mat Jais, Adiba Afreen

**Affiliations:** ^1^Department of Biomedical Sciences, Faculty of Medicine and Health Sciences, Universiti Putra Malaysia, 43400 Serdang, Selangor, Malaysia; ^2^Natural Products Research Laboratory, Anwarul Uloom College of Pharmacy, New Mallepally, Hyderabad, Andhra Pradesh 500001, India

## Abstract

The present study was conducted to evaluate the antiulcerogenic effect and recognize the basic mechanism of action of *Tabernaemontana divaricata* (L.) R. Br. flowers. *T. divaricata* flower methanolic extract (TDFME) was screened for antiulcer activity versus aspirin and ethanol induced gastric ulcers at three doses—125, 250, and 500 mg/kg—orally using misoprostol as a standard. Besides histopathological examination, seven parameters, that is, ulcer index, total protein, nonprotein sulphhydryls, mucin, catalase, malondialdehyde, and superoxide dismutase levels, were estimated. In addition to HPLC profiling, GC-MS analysis and electrospray ionization—high resolution mass spectral (ESI-HRMS) analysis of crude TDFME were carried out in an attempt to identify known phytochemicals present in the extract on the basis of *m/z* value. The results revealed a significant increase in the levels of catalase, superoxide dismutase, mucin, and nonprotein sulphhydryls, while they revealed a reduction in ulcer index, the levels of total protein, and malondialdehyde. Histopathological observations also demonstrated the protective effect. Though all the doses of TDFME exhibited gastroprotective function, higher doses were found to be more effective. Mass spectral analysis gave a few characteristic *m/z* values suggesting the presence of a few known indole alkaloids, while HPLC profiling highlighted the complexity of the extract. TDFME was found to exhibit its gastroprotective effect through antioxidant mechanism and by enhancing the production of gastric mucous.

## 1. Introduction

*Tabernaemontana divaricata* (L.) R. Br. ex Roemer and Schultes (Apocynaceae) has high medicinal value, and it is used in the ethnic systems of medicine all over the world. Traditionally, crepe jasmine flowers are used to treat corneal inflammation [1], ophthalmia [2, 3], and in Unani medicine for the management of pain [4]. Also the flowers soaked water is sprinkled on smallpox patients [5]. Recent investigations on *T. divaricata* flowers reported hippocratic screening [6], antianxiety [7], anticonvulsant [8, 9], antidiabetic and cytotoxic [10], antifertility [11], antiinflammatory [12, 13], antioxidant [13], and gastroprotective effects [14]. In continuation to our research on pharmacological assessment of the gastroprotective potential of *T. divaricata* flowers, the present study was conducted to understand its mechanism of action in chemically induced gastric ulcers.

## 2. Material and Methods

### 2.1. Plant Material and the Preparation of Extract

The pinwheel flowers were collected from the local areas of Ameerpet and Mallepally of Hyderabad district. Flowers of *Tabernaemontana divaricata* (L.) R. Br ex. Roemer and J. A. Schultes belonging to the Apocynaceae family were authenticated by a plant taxonomist, Dr. P. V. Prasanna (Scientist—E) at the Botanical Survey of India, Deccan Regional Centre, Hyderabad (establishment under the Ministry of Environment & Forests, Government of India). The specimen deposited in the herbarium was assigned a voucher number BSID 887. The extract was prepared as per the procedure described earlier [14]. Briefly, after collection, flowers were shade dried and coarsely powdered. Approximately 500 gm of the powdered flowers was extracted using methanol 99% pure (SD Fine-Chem) in a soxhlet apparatus. The extract was concentrated under reduced pressure and stored in an airtight container in a refrigerator at the temperature below 10°C. The dried mass of crude *Tabernaemontana divaricata *flower methanolic extract (TDFME) was found to be 34% w/w with respect to the dried flowers. The solution of TDFME was prepared using distilled water for the evaluation of the antiulcer activity. 

### 2.2. Drugs and Chemicals

Chloroform AR, diethyl ether, methanol AR, phenolphthalein pH indicator solution, and sodium hydroxide pellets were procured from SD Fine-Chem Limited, Mumbai, while pure aspirin was obtained from Divis Laboratories, Hyderabad. Absolute ethanol was purchased from Changshu Yangyuan Chem, China, and misoprostol (as misoprost-200) was purchased from Cipla Ltd., Goa, while surgical spirit was obtained from Kakatiya Pharma, Hyderabad. Topfer's Reagent and distilled water were obtained from Nice Chemicals, China, and Stangen Fine Chemicals, Hyderabad, respectively. All chemicals were used without further purification.

### 2.3. Animals

Adult male Wistar rats weighing 150–200 gm were used for the evaluation of antiulcer activity. The animals were maintained under standard laboratory conditions in polypropylene cages under 12 hr light/dark cycle, controlled temperature (24 ± 2°C), fed with commercial pellet diet, and water *ad libitum *in an animal house approved by the Committee for the Purpose and Supervision on Experiments on Animals (Reg. no. 1534/PO-/a/11/CPCSEA). All the animals were acclimatized to the laboratory environment for 10 days before the initiation of the experiments. The protocol (IAEC/AUCP/2011-12/02) was approved by the Institutional Animal Ethical Committee before the commencement of animal experimentation. All measures were taken to ensure that the experiments were conducted in accordance with the instructions of IAEC, Anwarul Uloom College of Pharmacy, New Mallepally, Hyderabad, Andhra Pradesh, India.

### 2.4. Acute Toxicity Test and Selection of Test Doses

Three test doses (125, 250, and 500 mg/kg) were selected in range of 1/16 to 1/4 of the maximum oral safe doses reported in earlier articles [7, 9, 11]. 

### 2.5. Evaluation of Antiulcer Activity by *In Vivo* Assays

#### 2.5.1. Acute Ethanol Induced Gastric Ulcers

All the animals (*n* = 6) in each group were fasted for 36 hours before the administration of ethanol. The standard drug (misoprostol 100 *μ*g/kg, p.o.) or the test extract was administered one hour before ethanol administration. Ethanol (90%) was administered to all animals at a dose of 1 mL/200 gm. After one hour all animals were sacrificed, and stomachs were isolated [15]. Lesion severity was determined by measuring ulcer index. Later, stomachs were subjected to the estimation of catalase, superoxide dismutase, malondialdehyde, mucin, total protein, and nonprotein sulphhydryl contents following standard procedures.

*(1) Ulcer Index (UI)*. Mean ulcer score for each animal is expressed as ulcer index. The stomachs were washed with running water to see the ulcers in the glandular portion of the stomach. The number of the ulcers per stomach were noted and the severity of the ulcers was scored microscopically with the help of hand lens (10x) and scoring was done as per Kulkarni (1999) [16] as following

 0 = normal stomach,
 0.5 = red colouration,
 1 = spot ulcers,
 1.5 = hemorrhagic streaks,
 2 = ulcers > 3 mm but <5 mm,
 3 = ulcers > 5 mm.

*(2) Assessment of the Oxidative Damage in Gastric Tissue.* After measuring the ulcer index, the stomachs were washed with 0.9% (w/v) NaCl, cut into small pieces, and homogenized with a glass homogenizer in ice-cold 0.15 M KCl solution to produce a 20% (w/v) homogenate. The homogenate was used for the determination of various biochemical parameters. 

*(a) Estimation of Catalase (CAT).* Catalase activity in stomach tissue was determined according to the method of Leyck and Parnham (1990) [17]. The stomach tissue was scraped and homogenized in ice cold saline medium with the help of a homogenizer. The solution was centrifuged for 10 minutes at 3000 g-force and collected for the experiment. 100 *μ*L of the supernatant was added to a solution of 3 *μ*L of H_2_O_2_, phosphate buffer mixture (50 mM phosphate buffer, pH 7.0, and 30% H_2_O_2_). The change in optical density at 240 nm per unit time was measured. 

*(b) Estimation of Superoxide Dismutase (SOD)*. Superoxide dismutase activity in stomach tissue was determined according to the method of Fridovich (1995) [18]. The stomach tissue was scrapped and homogenized in ice cold normal saline medium with the help of a homogenizer. Then, the tissue homogenate was centrifuged for 10 minutes at 3000 g-force and the supernatant was collected and used for the estimation of SOD activity. 10 mL of the solution was taken in a test tube and mixed with 0.5 mL of 50 mM phosphate buffer (pH 7.8), 0.1 mM of EDTA, 0.05 mM xanthine, and 0.01 mM cytochrome c, and then, 100 mL of 2.5 mM of xanthine oxidase was added to start the reaction, and the absorbance was measured at 550 nm. 

*(c) Determination of Malondialdehyde (MDA)*. The method reported by Utley et al. (1967) [19] was followed. The animals were killed 1 h after ethanol administration. The stomachs were removed and each was homogenized in 0.15 Mol/L KCl (at 4°C) in a homogenizer to give a 10% w/v homogenate. Aliquots of homogenate 1 mL in volume were incubated at 37°C for 3 h in a metabolic shaker. Then, 1 mL of 10% aqueous trichloroacetic acid (TCA) was added and mixed. The mixture was then centrifuged at 800 g-force for 10 min. One mL of the supernatant was removed and mixed with 1 mL of 0.67% 2-thiobarbituric acid in water and placed in a boiling water bath for 10 min. The mixture was cooled and diluted with 1 mL distilled water. The absorbance of the solution was then read at 535 nm. The content of malondialdehyde (nmol/g wet tissue) and index of the magnitude of lipid peroxidation were then calculated by referring to a standard curve of the malondialdehyde solution. 

*(d) Determination of Gastric Wall Mucin Content (GWM)*. Mucin content was estimated by the method described by Corne et al. (1974) [20] with some modifications. The glandular segments of the stomachs of rats subjected to the ethanol induced ulcers model were isolated and weighed. Each glandular segment was immediately immersed in 10 mL of 0.1% alcian blue solution (0.16 M sucrose/0.05 M sodium acetate, pH 5.8). After immersion for 2 h, excess dye was removed by two successive rinses with 10 mL of 0.25 M sucrose, first for 15 min and then for 45 min. The stomachs were all sequentially transferred to a 0.5 M magnesium chloride and shaken for 2 h. Four mL of the blue extract was then shaken vigorously with an equal volume of ether. The resulting emulsion was centrifuged at 3600 g-force and the absorbance of the aqueous layer was read at 580 nm. The amount of alcian blue extracted per gram of wet glandular tissue was then calculated. 

*(e) Estimation of Nonprotein Sulphhydryls (NPSH).* Gastric mucosal nonprotein sulphhydryls were measured according to the method of Sedlak and Lindsay (1968) [21]. The glandular part of the stomach was homogenized in ice-cold 0.02 mMol/L ethylenediaminetetraacetic acid (EDTA). Aliquots of 5 mL of the homogenates was mixed in 15 mL test tubes with 4 mL of distilled water and 1 mL of 50% w/v trichloroacetic acid (TCA). The tubes were shaken intermittently for 10 min and centrifuged at 3000 g-force. Two mL of supernatant were mixed with 4 mL of 0.4 mol/L Tris buffer at pH 8.9. Then 0.1 mL of 0.01 M 5,5′-dithiobis (2-nitrobenzoic acid) (DTNB) was added, and the volume was made up to 10 mL with 3.9 mL of absolute methanol. The sample was shaken, and the absorbance was measured within 5 min of DTNB addition at 412 nm against a reagent blank.

*(f) Estimation of Total Protein Content (TP)*. Total protein content was estimated by the method of Lowry et al. (1951) [22]. The dissolved proteins in gastric juice were estimated in the alcoholic precipitate obtained by adding 90% alcohol with gastric juice in a 9 : 1 ratio, respectively. Then, 0.1 mL of alcoholic precipitate of gastric juice was dissolved in 1 mL of 0.1 N NaOH. From this, 0.05 mL was taken in another test tube and 4 mL of alkaline mixture was added and allowed to stand. After 10 min, 0.4 mL of phenol reagent was added and again allowed to stand for 10 min for the development of colour. Reading was taken against a blank prepared with distilled water at 610 nm. The protein content was calculated from the standard curve prepared with bovine albumin and has been expressed in terms of *μ*g/mL of gastric juice. 

#### 2.5.2. Aspirin (NSAID) Induced Gastric Mucosal Damage

The gastric ulcers were induced by administering aspirin (200 mg/kg, p.o.) once daily for five days [23]. The animals were divided into five groups (*n* = 6). Group-I served as negative control and received only vehicle. Group-II served as standard and was treated with standard drug (misoprostol 100 *μ*g/kg, p.o.) [24]. Groups-III, -IV and -V received three different doses (125, 250 and 500 mg/kg) of test extract and served as test groups. All the treatments were made 30 min before administering aspirin once daily for five days. The rats were sacrificed on the fifth day, and the ulcer index was determined as mentioned earlier. 

*(1) Histopathological Studies. *The isolated stomachs were preserved in 15% formalin solution and were sent to the pathologist for histopathological examination by staining with haematoxylin and eosin. The morphological changes were observed and recorded with 100x lenses [25].

### 2.6. Analytical Profile of TDFME

#### 2.6.1. High Performance Liquid Chromatographic Analysis

HPLC analysis was carried out at the analytical development laboratory, Mylan Laboratories Limited, Bolarum, Hyderabad. HPLC analysis of TDFME was performed by gradient system using Waters 2996 photodiode array HPLC system with Kromasil C_18_- (250 × 4.5 mm, 5 *μ*m) column. Two solvents, A (water with 0.1% trifluoroacetic acid), and B (acetonitrile with 0.1% trifluoroacetic acid) were used for elution of constituents. The column was equilibrated in 85% A/15% B prior to the injection of the sample, and upon injection this composition was then changed to 60% A/40% B over 30 min utilizing a linear gradient followed by changing to 50% A/50% B over the next 10 min, and then, the concentration was returned to 85% A/15% B over the final 10 min. The flow rate was set to 1 mL/min, injection volume was 10 *μ*L, and column temperature was maintained at 30°C. The system was run for 60 minutes. The eluents were monitored at 255 and 350 nm. The peak numbers, retention times, areas, and percentage areas were recorded.

#### 2.6.2. Gas Chromatography—Mass Spectral Analysis

TDFME was subjected to GC-MS analysis by Agilent 6890 GC with 5973N MSD. CP-Sil 8 CB column of 30 m length × 0.25 mm id × 0.25 *μ* and hydrogen gas 1.2 mL/min was used as mobile phase. The sample was electro-ionized by a 70 eV source, and quadruple temperatures were maintained at temperature 230°C and 150°C, respectively. The transferline and injection temperature were 270°C. The oven temperature program was set as 50°C for 2 min initially, then as >10°C/min ramp with final temperature as 280°C held for 5 min.

#### 2.6.3. Electrospray Ionization—High Resolution Mass Spectral Analysis

Crude TDFME was subjected to high resolution mass spectral analysis by Thermo Exactive having a quadruple detector mass analyzer at the National Centre for Mass Spectrometry, the Indian Institute of Chemical Technology, Hyderabad, by Dr. U. V. R. Vijayasarthi. The sample was ionized by heated electro-spray ionization (HESI) method in positive mode. A mixture of methanol (80%) and formic acid (0.1%) was used as a mobile phase and the sample flow rate was set to 300 *μ*L/min. The capillary voltage was 4.0 kV and the source and transferline temperatures were 350°C and 300°C respectively. Nitrogen was used as an auxiliary and sheath gas, the supply was set to 35 and 10 units for respective purposes. The nitrogen was supplied through Peak Scientific NM30L nitrogen generator. The *m*/*z* values were recorded in the range of 0–1000. 

#### 2.6.4. Ehrlich's Confirmatory Chemical Test for Indole Alkaloids

For the detection of indole alkaloids, Ehrlich's reagent (0.25 g of p-dimethylaminobenzaldehyde dissolved in 130 mL sulphuric acid and 70 mL distilled water, allowed to stand for 24 hours) was used [26]. Test extract gave a blue colour indicating the presence of indole alkaloids. 

### 2.7. Statistical Analysis

Data obtained was analyzed by one-way ANOVA followed by Dunnett's multiple comparisons posthoc test using Graphpad Prism version 4.0, 32 bit for windows, Graphpad software, San Diego, California, USA (http://www.graphpad.com/). The values are expressed as Mean ± standard error of mean (SEM). *P* < 0.05 was considered statistically significant. 

## 3. Results and Discussion

### 3.1. Results of Ethanol Induced Gastric Ulcers Model

Ethanol is a common potential ulcerogenic agent that induces gastric hemorrhagic erosion on intragastric administration (Shetty et al., 2000) [27]. Ulcers caused by chemical inducers like ethanol are due to a number of contributing factors, which include effects on mucosal blood flow, platelet thrombi, damage to capillary endothelium and release of arachidonate metabolites, leukotriene C_4_/D_4_ (LTC_4_/D_4_) and platelet activating factor (PAF) (Goel and Bhattacharya, 1991) [28]. Rat gastric mucosal damage induced by high concentration of ethanol is widely used to investigate gastroprotective effect of medicinal plants (Zhu et al., 1997) [29]. Ethanol induced lesion formation is due to different factors like stasis of gastric blood flow contributing significantly to the development of hemorrhagic as well as necrotic aspects of tissue injury. Miller at al. (1985) stated that the free radicals produced during lipid peroxidation of cells and the cell membranes produce mucosal mast cell degranulation leading to the release of vasoactive mediators including histamine [30]. Histamine is responsible for the activation of adenyl cyclase which produces cyclic AMP which in turn activates the gastric proton pump and releases hydrogen ions (Tripathi, 2003) [31]. The amount of hydrogen ions generated is proportional to the gastric acid formed. When there is an increase in the gastric acid production, this leads to erosion or self-digestion of the gastric mucosa that protects the stomach. Thus, this may render that gastric mucosa more vulnerable to further damage or infections.

The products of 5-lipoxygenase pathway may also play a key role in the development of such ulcer (Lange et al. 1985) [32]. Mizui and Doteuchi (1986) and Terano et al., (1989) reported the involvement of free radicals in ethanol induced gastric ulceration and antioxidant action, and lipid peroxidation, respectively [33, 34]. Alcohol causes ulceration due to the excessive production of gastric mucosal LTC-4 and LTD-4 (Nielsen et al., 1987) [35]. Glavin and Szabo (1992) further explained the mechanism of ethanol induced gastric ulcers. They mentioned that ethanol induces ulcers by reducing the gastric mucosal blood flow and mucus production in the gastric lumen, decreasing endogenous glutathione and prostaglandin levels, and increasing ischemia, gastric vascular permeability, acid “back diffusion,” histamine release, efflux of sodium and potassium, influx of calcium, generation of free radicals, and the production of leukotrienes [36]. According to Al-Harbi et al., (1997) the genesis of ethanol induced gastric lesions is multifactorial with the depletion of gastric wall mucous and leukotriene release [37]. 

Oral administration of absolute alcohol causes severe damage to the gastric mucosa. The hemorrhagic and necrotic effects of ethanol can be reversed by prostaglandins as they effectively protect the mucosa against chemically induced gastric injuries (Robert et al. 1979) [38]. Therefore, treatment with analogous prostaglandins could be effective in reducing this type of damage. Considering the above fact, misoprostol (methyl-PGE_1_ ester) was used as the standard drug in the present study. The mechanisms involved in gastric mucosal protection of misoprostol are stimulation of gastric and duodenal mucosal production and bicarbonate secretion by the gastric mucosa, the increase in mucosal blood flow which maintains mucosal integrity, the efficient removal of toxic substances, the inhibition of basal gastric acid secretion, and in response to food, histamine, pentagastrin, and coffee by direct action on parietal cells [39–42]. A comparative study was conducted in rats to assess mucosal protection offered by misoprostol, cimetidine, and sucralfate against gastric ulcers induced by aspirin, indomethacin, stress, sodium taurocholate, and ethanol. Misoprostol offered better gastroprotection by reducing the mean number of gastric lesions by 58% and 70% of the control group at 100 and 200 *μ*g/kg, respectively. Further, it is stated that misoprostol provides mucosal protection by a mechanism that does not require a reduction of gastric acid secretion for its protective effect [43]. The results of ethanol induced gastric ulcers model are presented in Table 1.

#### 3.1.1. Ulcer Index (UI)

The standard drug, misoprostol 100 *μ*g/kg and test extract (TDFME) at all the test doses used in the study (125–500 mg/kg) showed significant reduction (*P* < 0.001) in ulcer index.

*(1) Catalase (CAT) and Superoxide Dismutase (SOD)*. The standard drug and the high dose of test extract significantly (*P* < 0.01) raised the level of catalase. Low and moderate doses of TDFME also produced a similar effect, but the level of significance found was *P* < 0.05. Misoprostol and test extract at moderate and high doses (250 and 500 mg/kg) used in the study produced significant increase in level of superoxide dismutase with *P* < 0.001 and *P* < 0.05, respectively, while there was no significant effect observed with the low dose of test extract (125 mg/kg). Das et al. (1997) reported that in stress-induced gastric ulceration, MDA levels significantly increase with the concomitant decrease in CAT and SOD concentrations [44]. Test extract at various doses in the present study significantly decreased MDA and increased CAT and SOD concentrations suggesting, possibility of antioxidant activity mediated gastroprotection.

*(2) Malondialdehyde (MDA)*. Misoprostol and TDFME at moderate and high doses produced a significant decrease (*P* < 0.001) in the level of malondialdehyde. Low test dose also limited MDA concentration with *P* < 0.01. Stress inactivates mucosal prostaglandin synthesis by accumulating hydrogen peroxide (prostaglandin biosynthesis inhibitor) and by generating reactive oxygen species (ROS) (Bandyopadhyay et al., 1999) [45]. The increase in MDA levels results in an increase in free radicals like superoxide anion, hydrogen peroxide and hydroxyl radicals. These radicals are responsible for cell degranulation by increasing the peroxidation of cell membrane lipids causing a loss of structural and functional integrity of cell membranes. Accumulation of hydrogen peroxide occurs in the mitochondria and cytosol which leads to an increase in lipid peroxidation if not scavenged by CAT (Michiels et al., 1994) [46]. Further, a strong relationship between the levels of gastric mucosal lipid peroxidation end products (marker of oxidative stress) and gastric ulceration in stress induced ulcers has been reported (Tandon et al., 2004) [47]. The decline in malondialdehyde (MDA) concentration indicates a decrease in lipid peroxidation, since it is the end product of lipid peroxidation (Buege and Aust, 1978) [48]. 

*(3) Gastric Wall Mucus (GWM)*. Dose dependent increase in mucin content was observed in test groups (with *P* value ranging from <0.05 to <0.001). The effect of the moderate test dose was comparable with that of the standard group. The maximum increase in mucin content was found to be in animals receiving high test dose, 500 mg/kg with (*P* < 0.001). Gastric mucous is an important protective factor for the gastric mucosa and consists of a viscous, elastic, adherent and transparent gel formed by 95% water and 5% glycoproteins that covers the entire gastrointestinal mucosa. Moreover, gastric mucous is capable of acting as an antioxidant and thus can reduce mucosal damage mediated by oxygen free radicals (Repetto and Llesuy, 2002) [49]. The mucous gel layer is a complex secretion containing inorganic materials, secretory IgA, lactoferin, high molecular weight proteins containing serine, threonine, proline and alanine and carbohydrates mainly galactose, galactosamine, glucosamine, and fructose. Approximately 600 carbohydrate side chains per molecule of glycoprotein are present (Schrager, 1969) [50]. The physicochemical properties make it a relatively resistant acid barrier (Flemstrom and Garner, 1982) [51]. Mucin is the major part of gastric mucous, an important pre-epithelial factor that acts as a first line of defense against ulcerogens (Zalewsky and Moody, 1979) [52]. Prostaglandins E_2_ and I_2_ are predominantly synthesized by the gastric mucosa and are known to inhibit the secretion of gastric acid and stimulate the secretion of mucous and bicarbonate. Hydrophobic surfactants like phospholipid secretion in the gastric epithelial cells are also stimulated by the prostaglandins (Aly, 1987) [53]. Decrease in mucosal secretion is thus considered important in gastric ulceration (Goel and Bhattacharya, 1991) [28]. The test extract and the standard drug showed similar effects, that is, increase in the production of mucin. The degree of enhancement observed in standard and test groups was also comparable. It would be reasonable to comment that TDFME exhibited prostaglandin analogous effect on gastric mucosa.

*(4) Nonprotein Sulphhydryls (NPSH)*. Treatment with the standard drug and TDFME 500 mg/kg produced significant restorative effect on nonprotein sulphhydryl concentration (with *P* < 0.001). TDFME low and medium dose (125 and 250 mg/kg) also elevated NPSH content. Sulphhydryl compounds in living organisms play a central role in the maintenance of gastric integrity, particularly when ROS are involved in the pathogenesis of tissue damage (Kimura et al., 2001) [54]. It is reported that a significant decrease in gastric non-protein sulph-hydryls occurs following ethanol administration by generating enormous amount of free radicals (Al Mofleh et al., 2007) [55]. Hiraishi et al., (1994) and Sener-Muratoglu et al., (2001) demonstrated that the increase in mucosal nonprotein sulphhydryl exerts a gastroprotective effect [56, 57].

*(5) Total Protein (TP)*. TDFME pretreatment at all the test doses significantly decreased protein content in gastric juice. 500 mg/kg test dose was more effective than the standard dose indicating strengthening potential on mucosal barrier and an increase in resistance against aggressive factors. Leakage of plasma protein into the gastric juice can cause weakening of the gastric mucosal barrier (Mizushima and Kobayashi, 1968; Grossman, 1978) [58, 59].

### 3.2. Results of Aspirin Induced Gastric Ulcers Model

**Figure 1:**
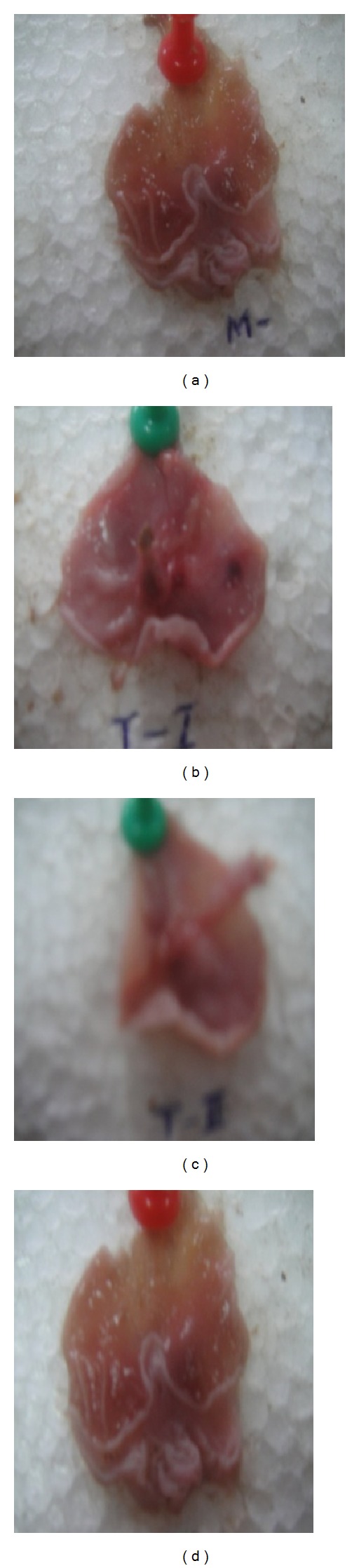
Photos of rat's stomachs subjected to aspirin induced gastric ulcers. Note: (a) standard (misoprostol 100 *μ*g/kg); (b) test-I (TDFME 125 mg/kg); (c) test-II (TDFME 250 mg/kg); (d) test-III (TDFME 500 mg/kg).

Aspirin induced gastric ulceration model showed dose dependent increase in ulcer protection. Test-I (TDFME 125 mg/kg) and Test-II (TDFME 250 mg/kg) significantly (*P* < 0.01) decreased the ulcer index while the standard and Test-III (TDFME 500 mg/kg) more significantly (*P* < 0.001) decreased the mean ulcer score (ulcer index). Thereby, the ulcer protective effect of high test dose (TDFME 500 mg/kg) was found to be more prominent than the standard (misoprostol). Results are shown in Figure 1 and Table 2.

#### 3.2.1. Histopathological Findings

**Figure 2:**
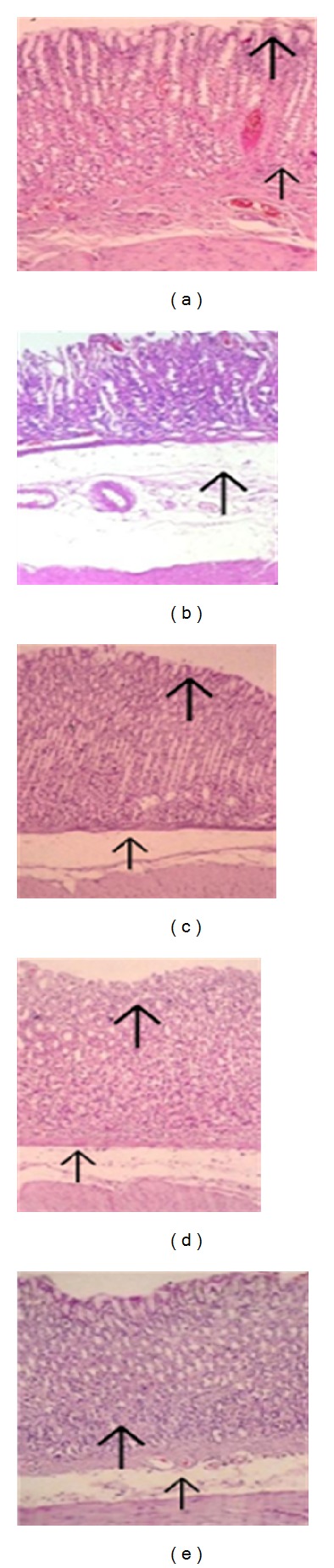
Histopathological slides of rat's stomachs subjected to aspirin induced gastric ulcers. Note: (a) negative control; (b) standard (misoprostol 100 *μ*g/kg); (c) test-I (TDFME 125 mg/kg); (d) test-II (TDFME 250 mg/kg) and (e) test-III (TDFME 500 mg/kg).

- *Negative Control.* Stomachs of the negative control group showed aggregates of inflammatory cells, mucosal ulceration, degenerated epithelial cells, and necrosis (thick arrow in Figure 2(a)). Severe oedema, inflammatory infiltration (macrophages and neutrophils), congestion in vascular spaces were observed in submucosa (Short arrow in Figure 2(a)). Results are shown in Figure 2.
- *Standard*. Mucosa of rats treated with the standard drug (misoprostol 100 *μ*g/kg) was found to be intact, while few scattered lymphocytes were present indicating inflammation (Thick arrow in Figure 2(b)).
- *TDFME* (125 mg/kg). TDFME (125 mg/kg) treated group revealed gastric ulceration and haemorrhage (haemorrhagic streaks) in mucosa (Thick arrow in Figure 2(c)). Submucosa showed moderate oedema and few scattered inflammatory cells (Short arrow in Figure 2(c)).
- *TDFME* (250 mg/kg). Mucosa of rats treated with TDFME (250 mg/kg) was found to be intact with few scattered lymphocytes (thick arrow in Figure 2(d)). Submucosa revealed mild oedema, congestion in vascular spaces (short arrow in Figure 2(d)).
- *TDFME* (500 mg/kg). Mucosa of rats treated with TDFME (500 mg/kg) was found to be normal (Thick arrow in Figure 2(e)). Submucosa showed mild oedema and scattered inflammatory infiltration (Short arrow in Figure 2(e)).

### 3.3. Results of HPLC Analysis

**Figure 3:**
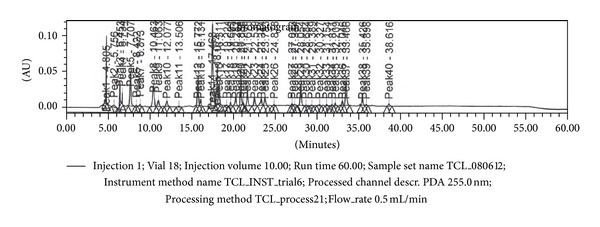
HPLC chromatogram of TDFME.

40 peaks and their respective retention times (RT) were recorded on HPLC analysis of TDFME as per the method described earlier. Peaks 5, 8, 12, 14, 15, 16, 20, 29, and 37 had a noteworthy area and percentage area. The results are shown in Figure 3 and Table 3. 

### 3.4. Results of GC-MS Analysis

**Figure 4:**
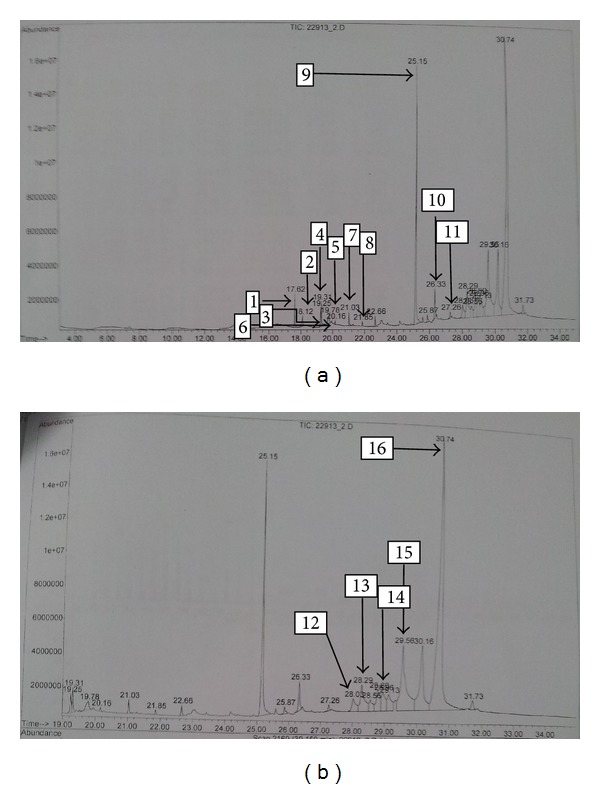
GC-MS spectra of TDFME.

The GC-MS analysis of TDFME reveals the presence of *α*-tocopherol (Vit. E), cycloartenol, docosane, eicosane, ergost-5-en-ol, ibogamine-18-carboxylic acid, 12-methoxy, methyl ester, 9-Octadecenoic acid, 11-Octadecenoic acid methyl ester, 9,12-octadecedienoic acid, palmitic acid, squalene, stigmasterol, tetracosane and urs-12-en-24-oic acid 3-oxo, methyl ester. The retention times, structures, molecular formulae and weights of compounds, quality match with Wiley 7N Database, and the percentage of the total composition is summarized in Table 4. A GC-MS spectrum showing corresponding peaks is presented in Figure 4. 

### 3.5. Results of Electrospray Ionization—High Resolution Mass Spectral Analysis

**Figure 5:**
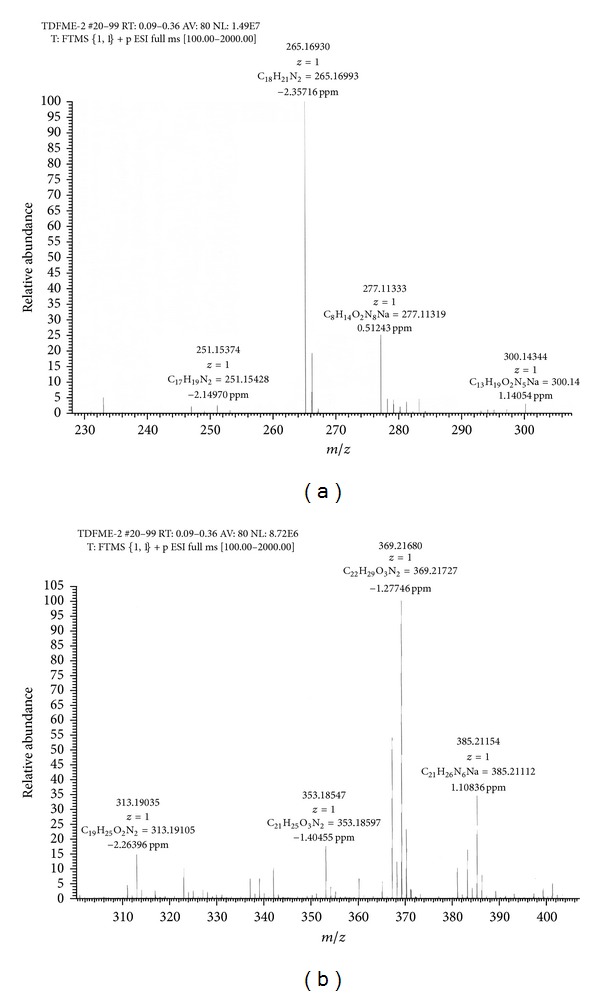
Electrospray ionization—high resolution mass spectra of crude TDFME.

A few characteristic *m*/*z* peaks were observed on ESI-HRMS analysis of crude TDFME. The recorded molecular weights and formulae were found to match with the reported indole alkaloids. Apart from corynanthean and ibogan indole alkaloids like 11-methoxy-N-methyldihydropericyclivine, 19-epivoacangine, isovoacangine, and isovoacristine, which have been reported earlier in the flowers (Arambewela and Ranatunge, 1991) [60], the observed [M + H]^+^ peaks in positive mode ESI-HRMS analysis reveal the probable presence of few more indole alkaloids belonging to subclasses aspidospermatan, corynanthean, ibogan, plumeran and miscellaneous sub-classes for the first time in the flowers. The compounds could be Apparicine, Voafinine, Vobasine, Voacangine, 3-Oxocoronaridine, 5-Oxocoronaridine, 6-Oxocoronaridine, 5-Hydroxyvoaphylline, Lochnericine, Pachysiphine and 3,14,4,19-tetrahydro-Olivacine or structural analogues with similar molecular formulae and weights. The observed [M + H]^+^ peaks and the list of probable compounds with their exact *m/z* values and molecular formulae as per the literature are summarized in Table 5. The *m/z* peaks and molecular formulae are shown in ESI-HR mass spectra in Figure 5. The members of Apocynaceae are rich in indole alkaloids, and in particular *T. divaricata* contains at least 66 characterized indole alkaloids (Pratchayasakul et al., 2008) [61]. The present study highlights the presence of certain uncharacterized indole alkaloids or their analogues in flowers that need to be elucidated with further exhaustive phytochemical studies and may possess medicinal properties and find application in modern medicine. 

Falcão et al. (2008) documented an extensive literature on antiulcer and antisecretory activities of various alkaloids [62]. Tabersonine is reported to be present in *T. divaricata* flowers (Gomez-Gonzalez et al., 1981) [63]. Tan et al. (2002) reported the antiulcer activity of Tabersonine isolated from *Enantia chlorantha *and *Voacanga africana *at a dose of 50 mg/kg orally in rats [64]. In addition to this, an indole alkaloid Tabernaemontanine isolated from flowers, leaves, stem bark, and roots of *T. divaricata* improved the blood supply to various organs and vascular tissue by vasodilation in dogs (Elkeiy et al., 1966; Van Beek et al., 1984) [5, 65]. This alkaloid may exhibit therapeutic effect in gastric injuries resulting from decreased blood flow to the gastric mucosa. Further, the crude extracts of *T. divaricata* are reported to possess antiinflammatory activity (Henriques et al., 1996; Satyanarayana et al., 2004; Thambi et al., 2006) [12, 13, 66]. According to the recent review report of Awaad et al., (2013) the prophylactic antiulcerogenic activity of natural products involves the antioxidant and antiinflammatory mechanisms while the therapeutic effects are due to the antisecretory and healing properties [67]. 

The present day treatment approach towards management of peptic ulcer disease employs antihistaminics (H_2_-receptor antagonists like cimetidine, famotidine, ranitidine, roxatidine) and anticholinergics (selective M_1_ muscarinic receptor antagonists like pirenzepine) that produce antisecretory and antispasmodic effects respectively (Miller, 1985) [30]. *T. divaricata* contains isovoacristine, an indole alkaloid that exhibited antihistaminic, anticholinergic, and skeletal muscle relaxant properties in a study on isolated guinea pig ileum and rabbit, respectively (Rao and Singri, 1979) [68]. Hence, the protective effect could be a consequence of antihistaminic and anticholinergic activities demonstrated by such phytochemicals. Besides these facts, the hippocratic *in vivo* screening of *T. divaricata* leaves and flowers conducted in rats by Taesotikul et al. (1989) suggests depressive effects on both peripheral and central nervous systems [6]. The depressant effect can limit the release of histamine, an important local hormone and chemical mediator that evokes gastric acid secretion with other stimulating factors like gastrin, vagal stimulation, and food by activating proton pump (H^+^K^+^ATPase) through H_2_ receptors (Tripathi, 2003) [31]. Therefore, the gastroprotective effect exhibited by *T. divaricata* flowers can be attributed mostly to the indole alkaloids present in it. 

Furthermore, a few indole alkaloids from *T. divaricata* have shown antimicrobial activity in *in vitro* studies. Apparicine isolated by cell suspension culture of *T. divaricata* inhibited *Polio III virus* at a concentration of 250 *μ*g/mL (Pawelka and Stöckigt, 1983; Andrade et al., 2005; Farnsworth et al., 1968) [69–71]. It exhibited antibacterial activity against *Corynebacterium, Escherichia, Proteus, Pseudomonas, Salmonella, Shigella,* and* Staphylococcus* in an *in vitro* study at a concentration of 1.2% (Rojas Hernández and Díaz Pérez, 1977) [72]. Voacamine found in bark, leaves, stem, and roots of *T. divaricata* by Karawya and Aboutabl (1979) demonstrated strong antimicrobial activity against gram positive bacteria such as *Bacillus subtilis* and *Staphylococcus aureus* and moderate activity against gram negative bacteria like *Escherichia coli* and *Pseudomonas aeruginosa* (Van Beek et al., 1984; Pratchayasakul et al., 2008) [5, 61, 73]. As mentioned in our previous article, *T. divaricata* extracts or its components can be effective against *Helicobacter pylori*. The biological activity of *T. divaricata* extracts against *H. pylori* is yet to be established [14]. 

Earlier *T. divaricata* was also studied for antioxidant effects by various investigators. Mandal and Mukherji (2001) demonstrated that *T. divaricata* is a good scavenger of air pollutants [74]. They also established that this plant has high level of antioxidative agents action like ascorbate peroxidase, catalase, glutathione, phenolic peroxidase, and superoxide dismutase. Further, Gupta et al. (2004) studied antioxidant and hepatoprotective effects of *T. divaricata* methanolic extract of leaves in carbon tetrachloride induced hepatotoxicity model. The extract produced significant dose dependent antioxidant and hepatoprotective effects by diminishing lipid peroxidation and elevating levels of antioxidant agents like Catalase, Glutathione and Superoxide dismutase [75]. Among the chief non-alkaloidal bioactive molecules is a flavonoid, the kaempferol, reported by Farooq et al. (1959) [76]. It exerts gastroprotective effect by scavenging stress generated oxygen metabolites and suppressing neutrophils infiltration in the inflammation that occurs during gastric ulceration (Borrelli and Izzo, 2000; Cheng and Koo, 2000) [77, 78]. The possibility of synergism between indole alkaloids and kaempferol cannot be ruled out.

## 4. Conclusion

It is concluded that *T. divaricata* (L.) R. Br. flower methanolic extract exhibits gastroprotective effect by enhancing the production of the gastric mucosa or preventing its depletion by aggressive factors while exhibiting antioxidant activity in chemically induced gastric ulceration in rats. The mass spectral analysis signifies the probable presence of few aspidospermatan, corynanthean, ibogan and plumeran sub-types of indole alkaloids in the flowers for the first time. The study also highlights the possible role of indole alkaloids in gastroprotective function of *T. divaricata*. 

## Figures and Tables

**Table 1:** Results of ethanol induced gastric ulcers model.

Parameters	Negative control	Standard misoprostol 100 *µ*g/kg	Test-I TDFME 125 mg/kg	Test-II TDFME 250 mg/kg	Test-III TDFME 500 mg/kg
Catalase	2.89 ± 0.11 (0.27)	4.09 ± 0.18 (0.44)^b^	3.86 ± 0.12 (0.49)^a^	3.94 ± 0.20 (0.49)^a^	4.23 ± 0.38 (0.94)^b^
Malondialdehyde	7.11 ± 0.30 (0.74)	2.69 ± 0.23 (0.57)^c^	5.64 ± 0.29 (0.73)^b^	4.94 ± 0.16 (0.40)^c^	3.58 ± 0.24 (0.60)^c^
Mucin content	0.52 ± 0.06 (0.15)	1.07 ± 0.11 (0.27)^b^	0.95 ± 0.15 (0.37)^a^	1.15 ± 0.10 (0.24)^b^	1.89 ± 0.11 (0.29)^c^
Nonprotein sulphhydryl concentration	2.31 ± 0.18 (0.45)	4.55 ± 0.17 (0.42)^c^	3.00 ± 0.19 (0.47)^b^	3.07 ± 0.09 (0.24)^b^	4.98 ± 0.00 (0.23)^c^
Superoxide dismutase	2.75 ± 0.35 (0.86)	5.99 ± 0.31 (0.78)^c^	2.44 ± 0.30 (0.74)^ns^	4.05 ± 0.23 (0.57)^a^	4.07 ± 0.24 (0.59)^a^
Total protein content	342.5 ± 4.09 (10.03)	313.2 ± 3.22 (7.91)^c^	321.7 ± 4.68 (11.47)^b^	316.5 ± 5.50 (13.49)^b^	251.7 ± 4.15 (10.17)^c^
Ulcer index	10 ± 0.28 (0.70)	4.50 ± 0.42 (1.04)^c^	7.08 ± 0.58 (1.42)^c^	4.00 ± 0.36 (0.89)^c^	1.50 ± 0.46 (1.14)^c^

Note: sample size (*n*) = 6 rats per group. Data is expressed as Mean ± Standard Error of Mean and Standard Deviation in parenthesis. ^a^ *P* < 0.05, ^b^ *P* < 0.01, ^c^ *P* < 0.001 and ^ns^nonsignificant versus negative control (on statistical analysis with ANOVA, followed by Dunnett's multiple comparison post-hoc test).

**Table 2:** Results of Aspirin induced gastric ulcers model.

Parameters	Negative control	Standard Misoprostol 100 *µ*g/kg	Test-I TDFME 125 mg/kg	Test-II TDFME 250 mg/kg	Test-III TDFME 500 mg/kg
Ulcer index	9.5 ± 0.42	4.00 ± 0.36^c^	7.16 ± 0.40^b^	7.5 ± 0.56^b^	1.50 ± 0.28^c^

^b^ *P* < 0.01, ^c^ *P* < 0.001.

**Table 3:** Results of the HPLC analysis of TDFME.

Peak number	Retention time	Percentage area
1	4.80	3.95
2	5.75	0.98
3	6.45	2.06
4	6.73	0.69
5	7.70	7.15
6	8.42	0.20
7	8.87	0.47
8	10.46	6.13
9	11.06	1.83
10	12.07	1.77
11	13.50	0.36
12	15.77	6.03
13	16.13	1.09
14	17.47	19.91
15	18.07	7.68
16	18.31	4.07
17	19.22	0.22
18	19.68	0.68
19	20.28	2.22
20	20.88	6.56
21	21.21	0.32
22	21.67	3.19
23	22.53	2.17
24	23.34	2.01
25	23.78	2.14
26	24.87	0.18
27	27.02	0.28
28	27.33	0.18
29	28.05	4.56
30	28.68	0.19
31	29.38	0.20
32	30.33	0.32
33	31.15	0.34
34	31.81	0.24
35	32.20	0.55
36	33.04	0.79
37	33.46	5.80
38	35.42	1.55
39	35.89	0.25
40	38.61	0.69

**Table 4:** Results of the GC-MS analysis of TDFME.

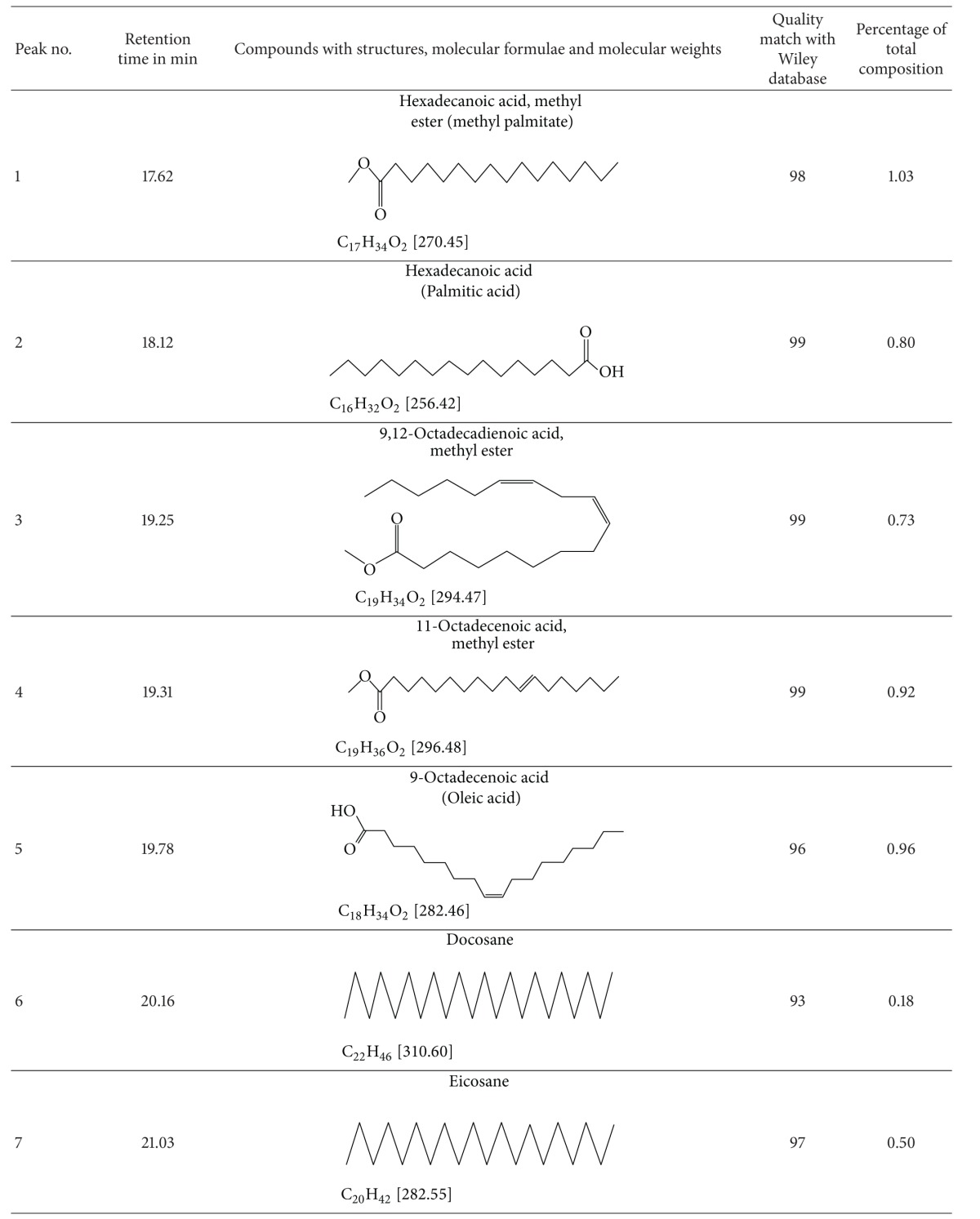 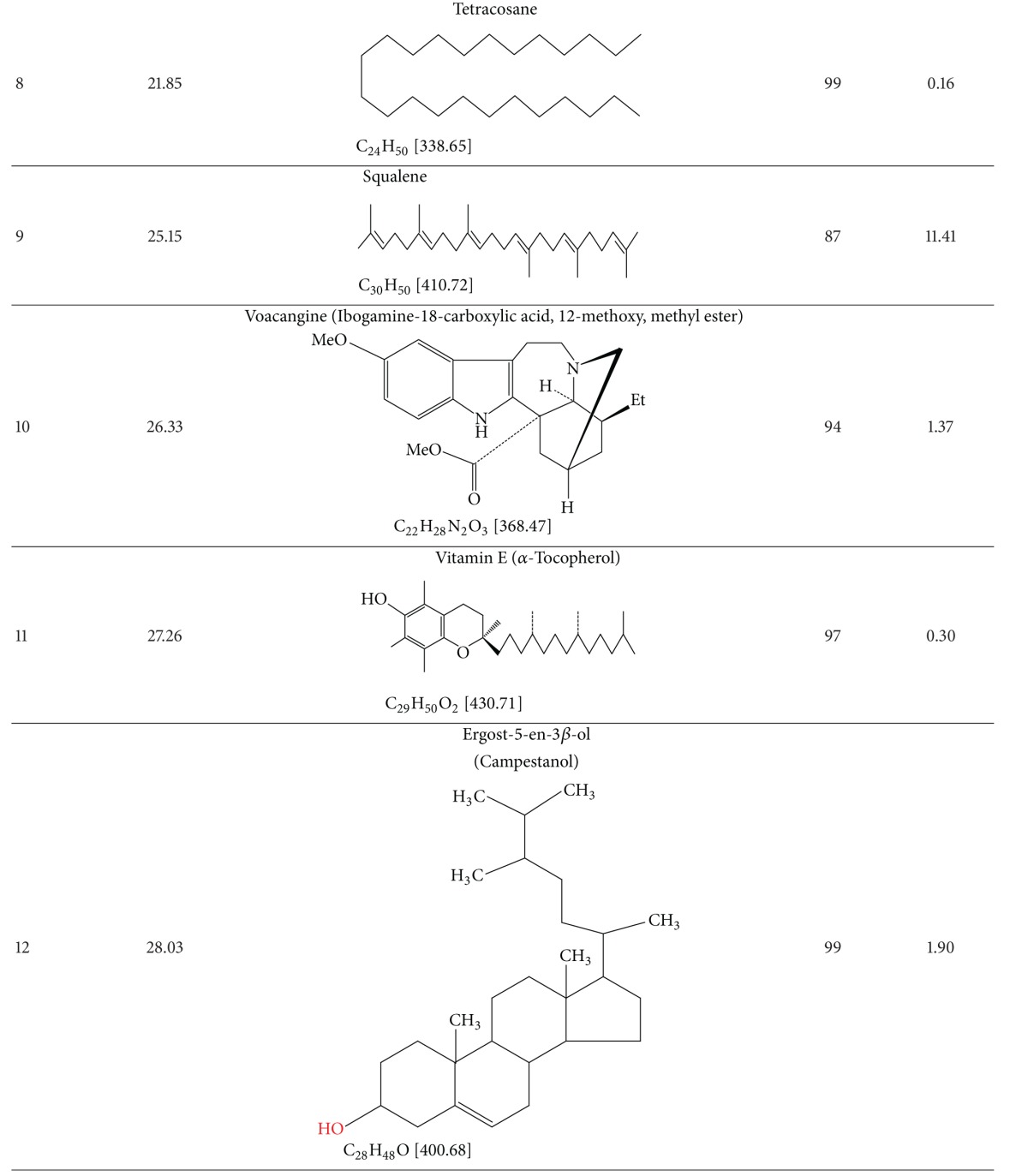 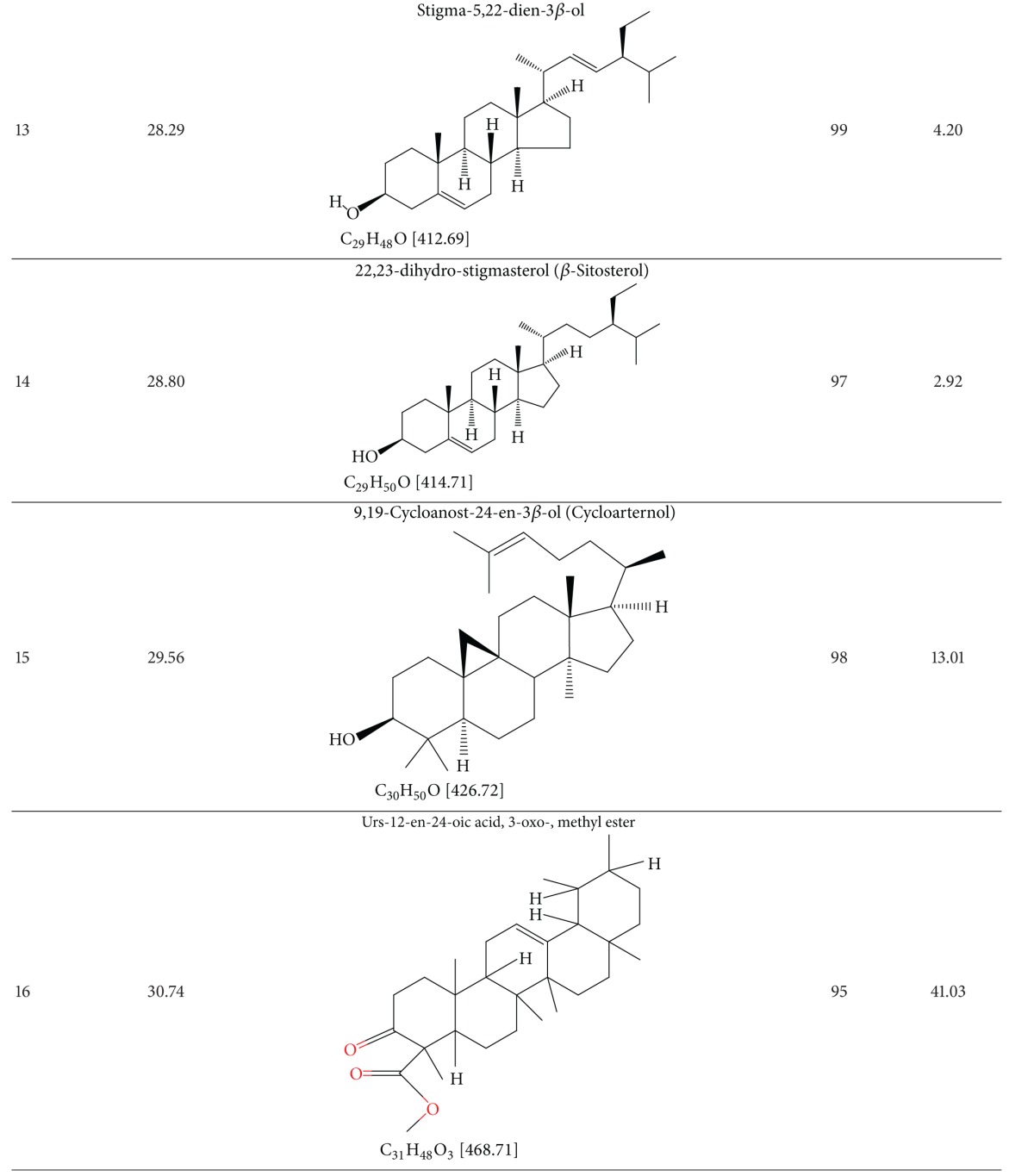

**Table 5:** Mass spectral data of Crude TDFME.